# Boosting tribo-mechanical, thermal expansion and electrical performance of waste polytetrafluoroethylene using high-strength carbide ceramics

**DOI:** 10.1038/s41598-025-32356-5

**Published:** 2026-01-13

**Authors:** Ahmed Kamal, Ahmed Gaafer, Ahmed O. Mosleh, Mohammed A. Taha

**Affiliations:** 1 Mechanical Engineering Department, Shoubra Faculty of Engineering, Benha University, 108 Shoubra St, Banha, Egypt; 2 Solid State Physics Department, National Research Centre, El Buhouth St, Dokki, 12622 Giza Egypt

**Keywords:** PTFE waste, Recycling, CTE, Mechanical properties, Electrical conductivity, Fraction coefficient, Powder metallurgy, Engineering, Materials science, Nanoscience and technology

## Abstract

Polytetrafluoroethylene (PTFE) is widely used across various industrial and technological fields, including aerospace, automotive, electronics, and chemical processing. This work presents a sustainable approach to reusing PTFE waste and enhancing the limited applications of PTFE, which are constrained by its inferior mechanical properties, thermal expansion, and wear resistance. The reuse of PTFE scrap produced by lathe shops improves its previously mentioned properties by adding high-strength and high-stability ceramics. In this context, powder metallurgy technology produces PTFE-based composites reinforced with boron carbide (B_4_C) nanoparticles and graphene nanosheets. The phase composition and microstructure were determined using X-ray diffraction (XRD) and field emission scanning electron microscopy (FESEM) techniques. XRD indicated the presence of PTFE and B_4_C phases in the chart of the specimens and the absence of graphene nanosheets due to their small quantity. The hybrid composite PTFE/5vol.% B_4_C/1 vol% graphene (GB6) recorded an increase in the microhardness, compressive stress, and Young’s modulus of 138.55%, 114.86%, and 78.70%, respectively, relative to the PTFE matrix (GB0). The results also indicated that the addition of reinforcements led to a significant improvement in wear resistance and thermal stability, with the GB6 sample recording a decrease in wear rate, coefficient of friction, and thermal expansion coefficient (CTE) value of 40.73%, 42.49%, and 57.94%, respectively, compared to the CB0 sample. Moreover, the addition of reinforcements to the PTFE matrix has the positive effect of increasing the electrical conductivity.

## Introduction

The production of polymers has long been accompanied by the difficulty of finding suitable uses for them after they are used. An enormous challenge arises from the recycling industry’s sluggish progress: the annual disposal of tens of millions of tons of obsolete polymeric materials. It causes environmental issues and, by extension, society^1–3^. Landfilling is becoming less appealing as a waste management option due to its lack of sustainability, rising costs, and shrinking area. It has been illegal since 1990 for ships to dump at sea. Furthermore, unsustainable derived processes remove many resources from the economic cycle. Because of the current low market price of waste plastics as starting materials, recycling may be an environmentally friendly and financially advantageous solution to the first two issues^4,5^.

Pure PTFE has restricted applicability as an engineering material because of its inadequate thermal dimensional stability, wear resistance, mechanical properties, and susceptibility to cold flow. The constraints can be mitigated by incorporating appropriate ceramic particles into a PTFE matrix to create PTFE-based composites^6–9^. PTFE is frequently utilized as a matrix in composite materials for equipment and machinery, including interacting mating surfaces in relative motion, such as bearings, seals, valves, and tribosystems, which are particularly significant due to its unique antifriction characteristics and exceptional chemical and frost resistance^10–12^.

Extensive research has been conducted to improve PTFE’s mechanical and electrical properties and frictional performance by adding ceramics. For example, Ronghao et al.^13^ looked at how plasma-treated carbon nanotubes affected the mechanical characteristics of PTFE/CF composites. Research indicates that adding a combination of fillers to PTFE improves its mechanical qualities by 8% compared to mono-modifier-only composites. Zhang et al.^14^ investigated composite materials of PTFE filled with aluminium oxide, carbon, and aramid fibres. It has been shown that adding small amounts of aluminium oxide to aramid fibres improves their mechanical and tribological characteristics by bout 10% and 13%, respectively. Fuchuan Luo et al.^15^ filled PTFE with glass fibre and Na1/2Sm1/2TiO_3_ to increase the electrical conductivity of PTFE composites. Ying Yuan et al. created a kind of PTFE composite material loaded with Si_3_N_4_. The electrical and thermal conductivity of the PTFE composite material improves with increasing Si_3_N_4_ concentration^16^. Recently, much focus has been directed on graphene owing to its remarkable attributes, including superior mechanical characteristics, low wear rate, and exceptional electrical^17,18^. B_4_C is undoubtedly one of the most important ceramic materials, possessing many valuable properties, including excellent oxidation resistance, high hardness, remarkable chemical attack resistance, low thermal expansion, and a melting point of approximately 2450 °C^19,20^. It is worth noting that powder metallurgy is an important technique to prepare composites based on metals^21–23^, ceramics^24,25^, or polymers^10,26–28^ to improve the desired properties due to the uniform distribution and refinement of reinforcement particles between bases. Producing PTFE-based composites with ceramic particle reinforcements has never been easier than with powder metallurgy. This method aids in maintaining PTFE’s thermal stability and chemical integrity by allowing for consistent reinforcement dispersion, exact composition control, and consolidation at comparatively low processing temperatures. In comparison to composites made using traditional melt processing techniques, powder metallurgy produced composites are more dense and homogeneous, and they exhibit better mechanical and tribological performance^29–32^.

Based on the above, various studies have studied enhancing mechanical properties and wear resistance. Still, this manuscript is unique in using the powder metallurgy technique to get composites based on PTFE waste from lathe shops, which have superb microhardness, coefficient of friction, and thermal expansion, improved by small percentages of hybrid graphene and B_4_C. Then, composites based on PTFE waste were prepared with proportions of mono and hybrid B_4_C up to 5 vol% % and graphene up to 1 vol% %. Additionally, it was examined how adding ceramics affected the manufactured composites’ microstructure, densification, CTE value, microhardness, elastic moduli, fraction coefficient, and electrical conductivity.

## Materials and methods

### Materials

The matrix material utilized in this study was PTFE waste, and the reinforcements were B_4_C nanopowders and graphene nanosheets. PTFE debris was extracted from industrial detritus, cleansed, desiccated, and powdered into fine particles.

### Morphology of renforcements

**Fig. 1:**
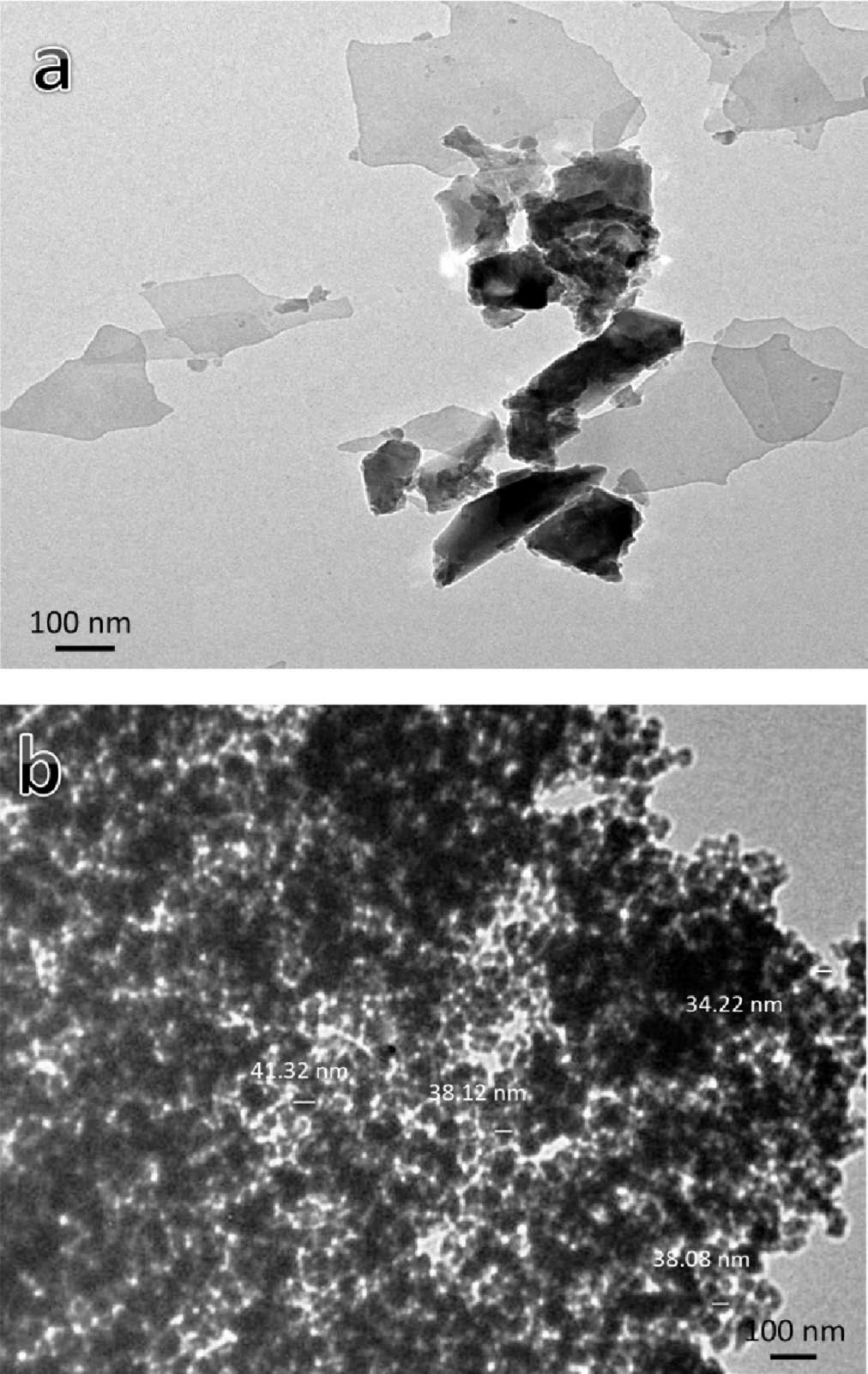
TEM micrographs of (**a**) graphene nanosheets and (**b**) B_4_C nanoparticles.

The morphological features (shapes and sizes) of milled powder were examined by transmission electron microscopy type JEOL JEM-1230. As illustrated in Fig. 1a, the graphene powder is observed as sheets by TEM, whereas the B₄C powders are observed as particles with an average size of 37.94 nm and a greater number of agglomerates, as shown in Fig. 1b.

### Samples preparation

**Fig. 2:**
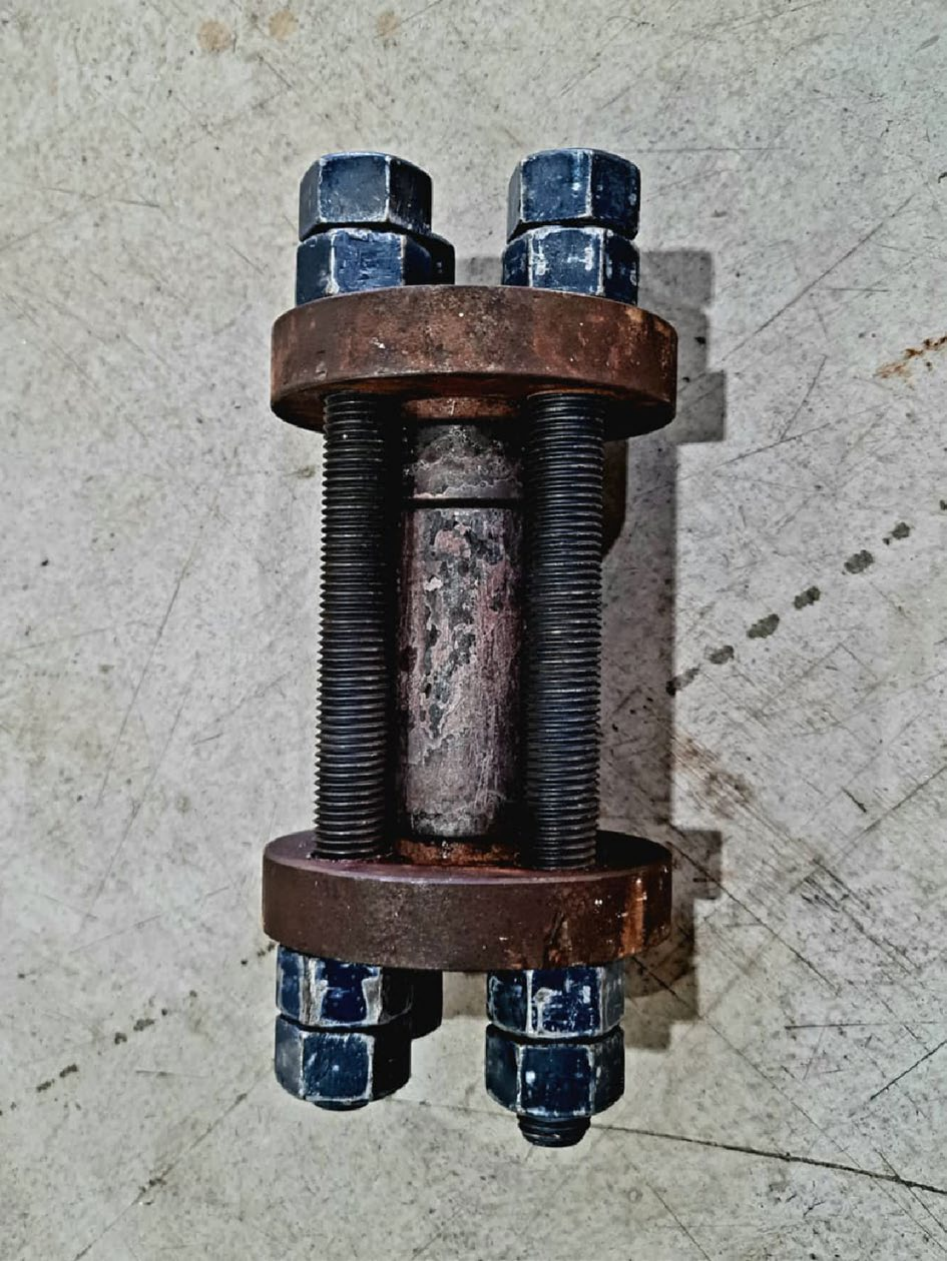
The image of a mold assembly.

**Fig. 3:**
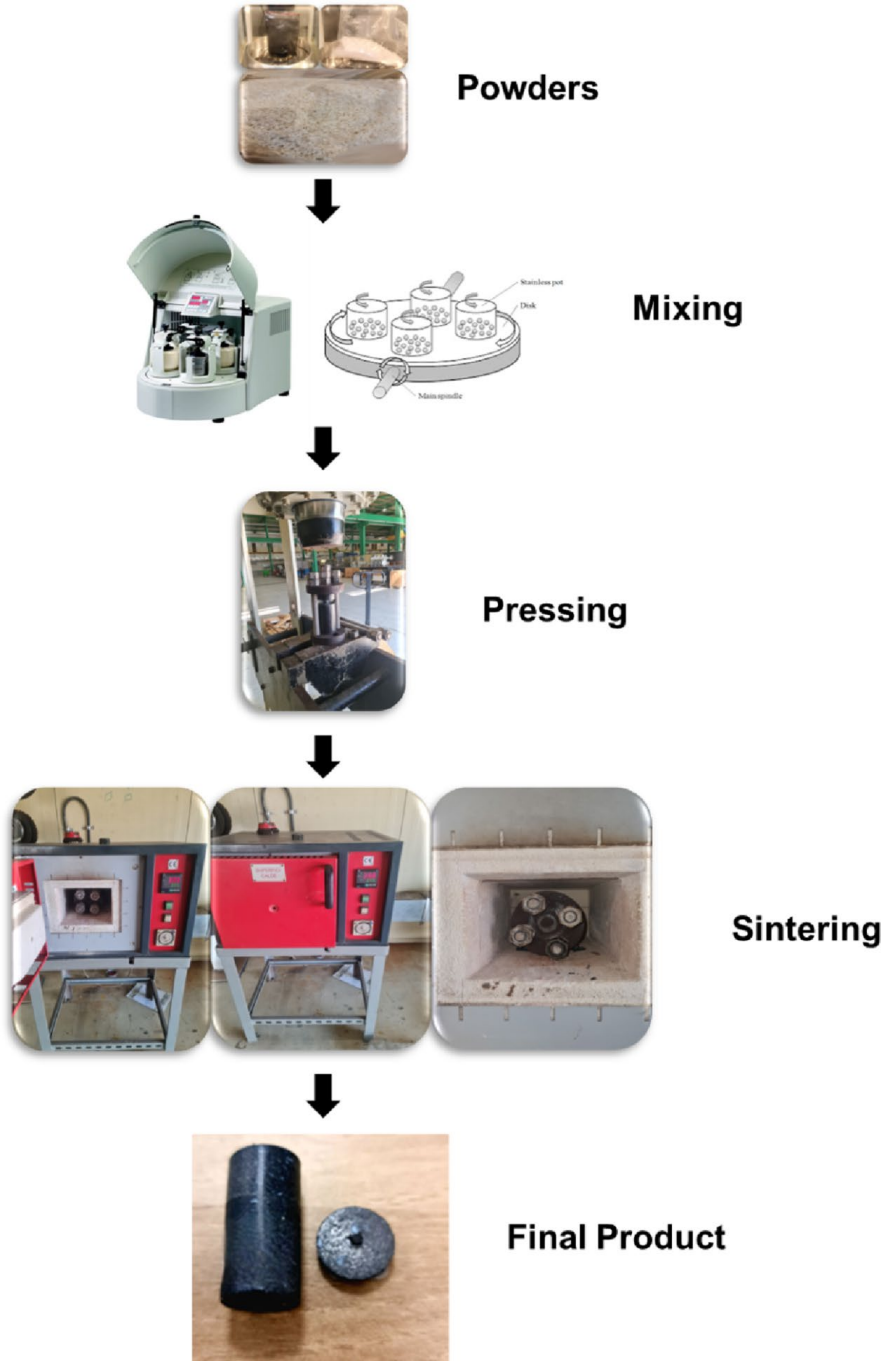
Schematic process of preparing samples.

**Fig. 4:**
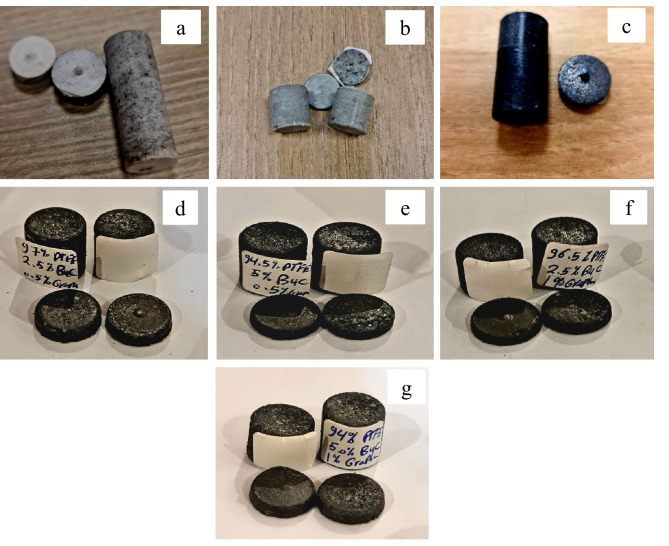
Images of prepared samples, (**a**) GB0, (**b**) GB1, (**c**) GB2, (**d**) GB4, (**e**) GB5, and (**f**) GB6.

The raw materials, which consisted of PTFE, graphene sheet, and B_4_C nanoparticles, were combined in a ball mill for 10 h at a ball-to-powder ratio (BPR) of 1:5 and a rotation speed of 120 rpm. The sample mixing process is carried out using a container and balls from an Al₂O₃. The mixing process without any process control agents (PCA) or lubricants being applied. The volumetric compositions of each sample are indicated in Table 1, which summarizes the batch design of the prepared composites. The mold assembly, depicted in Fig. 2, comprises numerous fundamental components engineered to endure the high temperatures and pressures inherent in the composite manufacturing process. This design guarantees the uniform pressure distribution and the proper ventilation of confined gases during the sintering process, thereby minimizing defects in the final composite, such as cavities or fractures. The mixture samples was loaded into a steel cylindrical die with an internal diameter of 20 mm and a height of 80 mm and compressed using a steel punch that applied a uniaxial load vertically. The pre-compacted samples were sintered in a programmable furnace at 370 °C for 1 h under 120 bar pressure, with heating and cooling rates of 3 °C/min. Figures 3 and 4 illustrated the preparing samples and the prepared samples, respectively.

**Table 1:** Batch design of the investigated prepared nanocomposites.

Samples name	Composition (Vol.%)		
	PTFE	B_4_C	graphene
GB0	100	0	0
GB1	99	0	1
GB2	95	5	0
GB3	97	2.5	0.5
GB4	96.5	2.5	1
GB5	94.5	5	0.5
GB6	94	5	1

### Characterization of sintered samples

#### Morphology and phase composition

The XRD (Philips PW) analysis was used to identify the phases of the sintered samples. The microstructure of sintered samples was investigated using FESEM in conjunction with energy dispersive X-ray analysis (EDX) (type Quanta FEG250, which has an accelerating voltage of 30 kV and a magnification of 10 × up to × 300,000).

#### Density and porosity

The bulk density (BD), total porosity (TP), and relative density (RD) of PTFE and all composite samples were investigated according to Eqs. 1, 2, and 3 using the Archimedes method (ASTM: B962-13).

1 $$\:\mathrm{B}\mathrm{D}=\:\frac{{\mathrm{W}}_{\mathrm{a}}-{\mathrm{W}}_{\mathrm{w}}}{{\mathrm{W}}_{\mathrm{w}}}\:\mathrm{x}\:{{\uprho\:}}_{\mathrm{w}}\:$$

2 $$\:\mathrm{T}\mathrm{P}=\left(1-\frac{\mathrm{B}\mathrm{D}}{\mathrm{T}\mathrm{D}}\right)\mathrm{x}100$$

3 $$\:RD=\:\frac{BD}{TD}\:x\:100$$

Where W_a_, W_w_, ρ, and TD are the weight of the sample in air, weight of the sample in water, density of water, and theoretical density of the sample calculated with the mixture rule, respectively. The bulk density, relative density, and total porosity are repeated three times, and the average values are calculated.

#### Thermal properties

Using rectangular bars, the Netzsch DIL402 PC examined the thermal expansion of samples between 25 and 200 °C at a heating rate of 5 °C/min. The CTE value is calculated as the slope of the straight line, derived from the relationship between thermal expansion and temperature.

#### Mechanical investigation

Vickers microhardness (Hv) of sintered samples was determined using this Eq. (4) with a load (p) of 1.9 N for 5 s^33^. In short, an indentation was performed using a measuring microscope, a video monitor, and a square pyramidal diamond with a 136° facet angle. Compute the hardness by dividing the applied force by the hierarchical contact area of the indenter.

4 $$\:\mathrm{H}\mathrm{v}=1.854\:\mathrm{x}\:\frac{\mathrm{P}}{{\mathrm{d}}^{2}}$$

Where d is the indentation diagonal.

The compressive strength (σ) was ascertained through uniaxial compression testing, with the samples having a diameter of 20 mm and a height of 15 mm in compliance using a strain rate of 1 mm/min by applying the subsequent formula:

5 $$\:{\upsigma\:}=\:\frac{\mathrm{F}}{\mathrm{A}}$$

F represents the load at fracture, and A represents the disc surface area.

The pulse-echo technique equipment measured the ultrasonic longitudinal (V_L_) and shear wave velocities (V_S_) in the PTFE and its composites. The following is how V_L_ and V_S_ are used to determine the values of Lame’s constants^34,35^.

6 $$\:{\uplambda\:}={\uprho\:}({V}_{L}^{2}-2{\mathrm{V}}_{S}^{2})$$

7 $$\:{\upmu\:}\:={\uprho\:}{V}_{S}^{2}\:$$

The following formula was used to determine the PTFE and its composites’ longitudinal modulus (L), Young’s modulus (Y), shear modulus (G), bulk modulus (B), and Poisson’s ratio (ν)^24,36^:

8 $$\:L=\lambda\:+2\mu\:$$

9 $$\:G=\mu\:$$

10 $$\:E=\mu\:\frac{3\lambda\:+2\mu\:}{\lambda\:+\mu\:}$$

11 $$\:B=\lambda\:+\frac{2}{3}\mu\:$$

12 $$\nu=\frac{\lambda\:}{2(\lambda\:+\mu\:)}\:$$

Microhardness was measured five times, while the group of elastic moduli and compressive strength were measured three times, and the average was calculated.

#### Tribology investigation

A TNO tester was used for the pin-on-disc wear test (Delft, The Netherlands). The wear test’s process conditions included a sliding period of 300 s, a speed of 0.25 m/s, and various applied loads of 5, 10, and 20 N. The following formulas determine the wear rate (WR) of sintered samples:

Weight loss = weight before wear - weight after wear (13)

14 $$\:\mathrm{W}\mathrm{e}\mathrm{a}\mathrm{r}\:\mathrm{r}\mathrm{a}\mathrm{t}\mathrm{e}=\:\frac{\mathrm{W}\mathrm{e}\mathrm{i}\mathrm{g}\mathrm{h}\mathrm{t}\:\mathrm{l}\mathrm{o}\mathrm{s}\mathrm{s}}{\mathrm{S}\mathrm{l}\mathrm{i}\mathrm{d}\mathrm{i}\mathrm{n}\mathrm{g}\:\mathrm{t}\mathrm{i}\mathrm{m}\mathrm{e}}$$

The ware test is repeated three times, and the average values of weight loss, ware rate and fraction coefficient are calculated.

#### Electrical conductivity

Using a Keithley 6517B system, the electrical conductivity (σ) of the sintered PTFE and its composites was determined at room temperature (28 °C) using the following formula:

15 $$\:\sigma\:=\:\frac{h}{RA}$$

R, h, and A represent the specimen’s electrical resistance, diameter, and surface area. The electrical conductivity is repeated three times, and the average value is calculated.

**Fig. 5:**
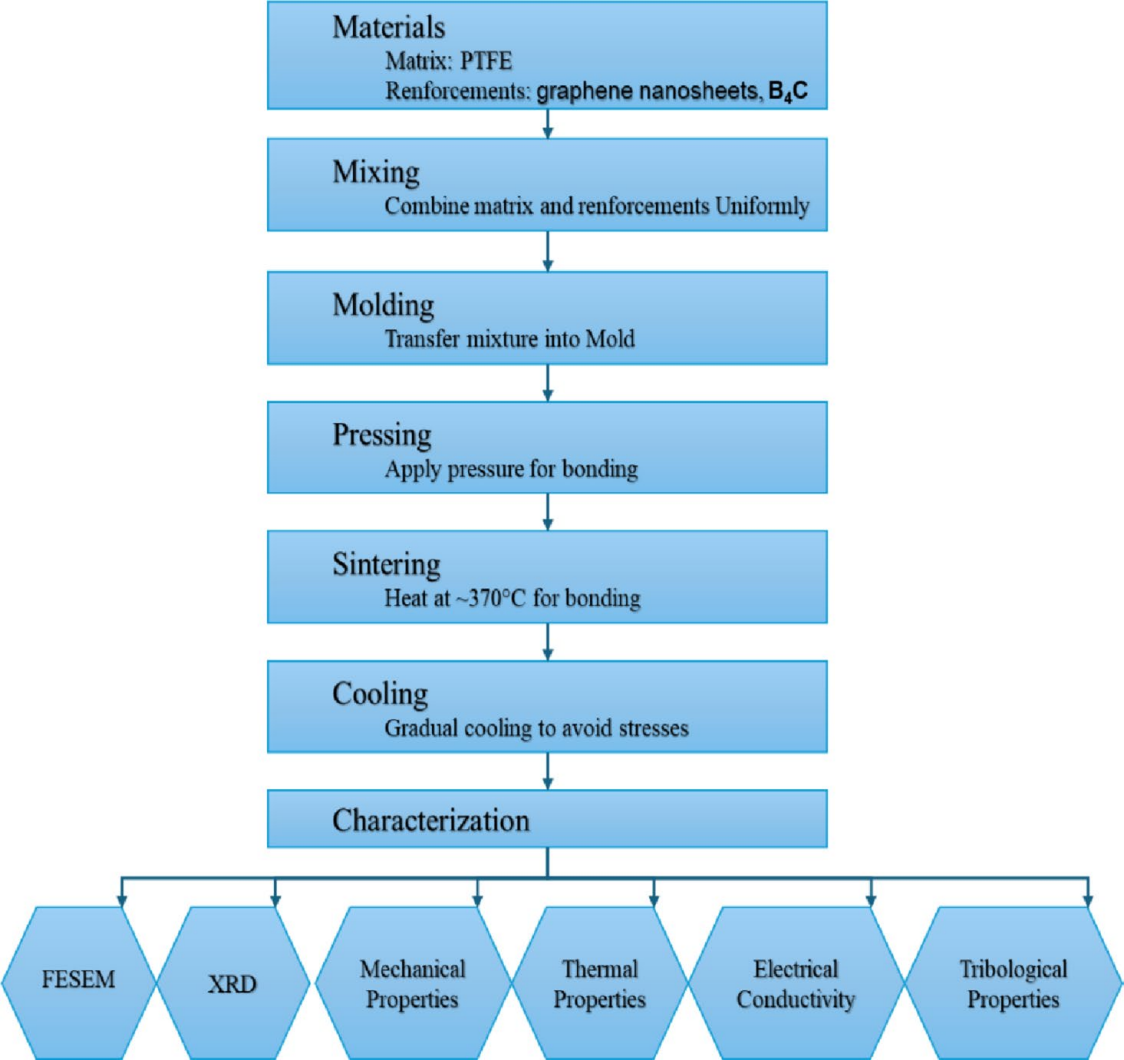
Schematic of methodology for PTFE composite fabrication and characterization.

The schematic procedure for the fabrication of PTFE matrix composite is shown in Fig. 5.

## Results and discussion

### Phase composition

**Fig. 6:**
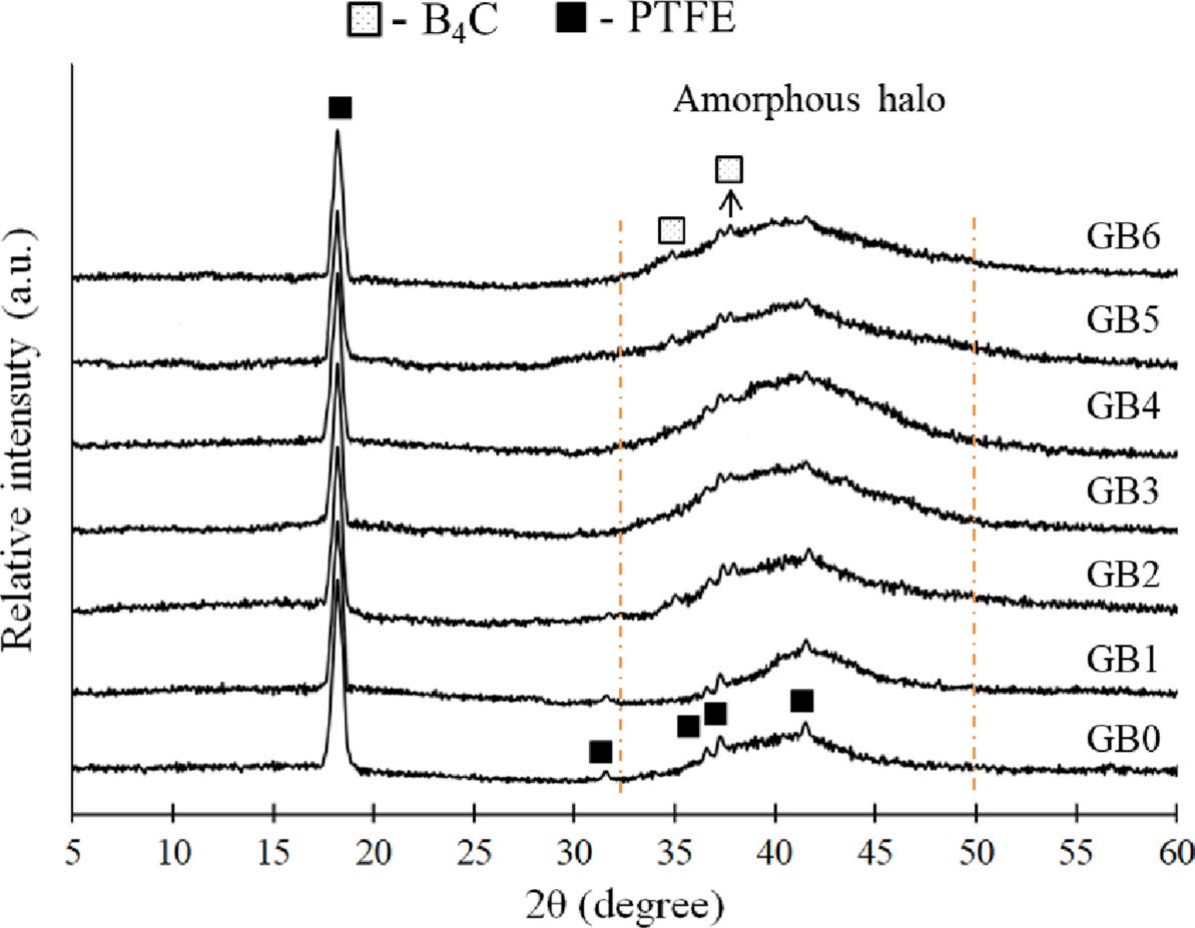
XRD patterns of prepared samples containing mono and hybrid graphene and B_4_C reinforcements.

The XRD method is an invaluable tool for evaluating the phase composition of the synthesized composites. Figure 6 illustrates the XRD patterns of sintered PTFE-based composites reinforced with mono and hybrid graphene sheets and B_4_C nanoparticles. X-ray spectra of pure PTFE exhibit two kinds of reflections: the crystalline and amorphous phases of PTFE. The JCPDS 47–2217 standard cards indicate the presence of diffraction peaks at 2θ = 18.10°, 31.60°, 36.64°, 37.13°, and 41.41°, besides an amorphous halo about 40°. In all composite samples, B4C nanoparticles are seen in phase at 2θ = 37.82° and 34.96°, as per JCPDS 35–0798. The distinctive peaks of graphene nanosheets were absent in the XRD pattern owing to their minimal quantities.

### Density and porosity

**Fig. 7:**
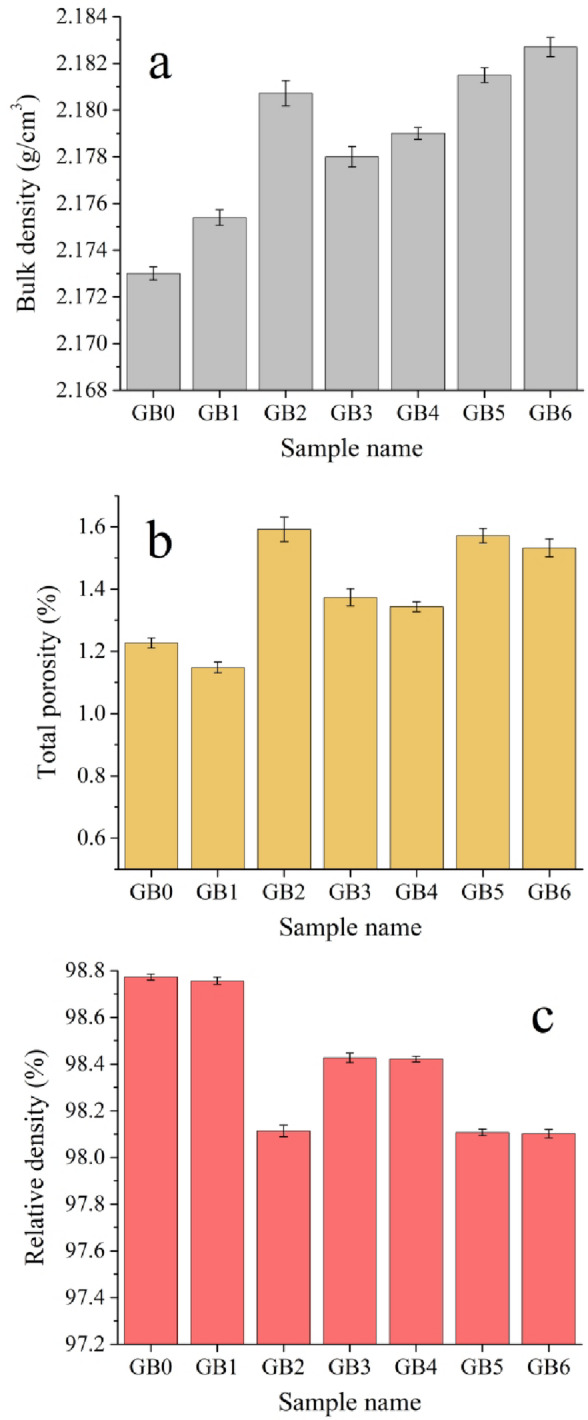
(**a**) Bulk density, (**b**) total porosity, and (**c**) relative density of PTFE matrix composites.

Figure 7; Table 2 illustrate the BD, TP, and RD of GB0, GB1, GB2, GB3, GB4, GB5, and GB6, sintered for one hour at a pressure of 120 bar and a temperature of 370 °C. The findings indicate that BD and TP of all composite samples’ somewhat exceed PTFE’s. Incorporating mono and hybrid ceramics into the PTES matrix results in a marginal increase in BD and TP of the composites. The TD value of GB0, GB1, GB2, GB3, GB4, GB5, and GB6 samples is 2.200, 2.175, 2.181, 2.178, 2.179, 2.182, and 2.183 g/cm³, respectively. Comparing the TD and BD yields the RD of all samples; the RD of GB0 (99.001 g/cm³) marginally falls to 98.773, 98.761, 98.408, 98.436, 98.426, 98.130, and 98.125 g/cm³, respectively. This increase in bulk density is due to the greater densities of graphene and B_4_C relative to pure PTFE, resulting in an increased mass per unit volume. The existence of agglomerates, inadequate interfacial bonding, and microvoids generated during processing impede the full densification of the composite. As a result, although the bulk density rises with the incorporation of denser reinforcing phases, the RD diminishes due the addition of reinforcements negatively affects the compactness of the PTFE matrix and prevents the particles from aligning as tightly as PTFE, thus increasing the total porosity. However, its RD value decreases because the material becomes less compact than its theoretical fully dense counterpart^37,38^.

**Table 2:** Effect of reinforcement addition on BD, RD, and TP of sintered samples.

Samples name	BD (g/cm^3^)	RD (%)	TP (%)
GB0	2.173	98.77	1.227
GB1	2.175	98.85	1.148
GB2	2.181	98.41	1.592
GB3	2.178	98.63	1.374
GB4	2.179	98.66	1.343
GB5	2.182	98.43	1.572
GB6	2.183	98.47	1.532

### Microstructure

**Fig. 8:**
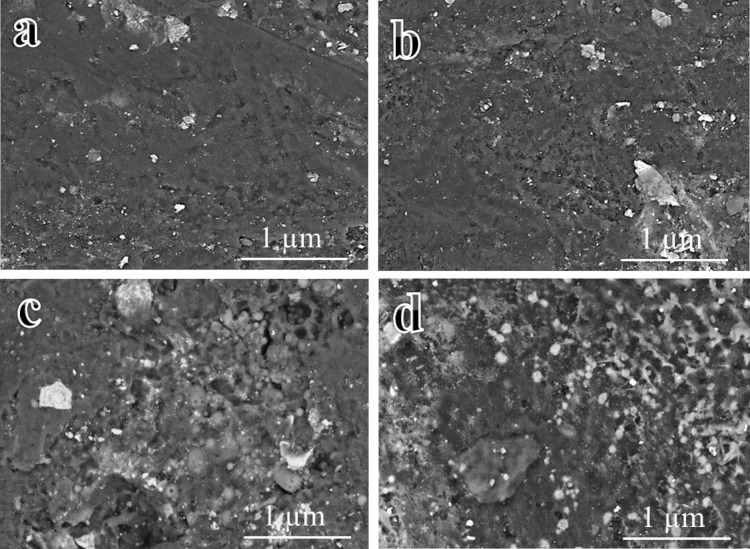
FESEM images of sintered (**a**) GB0, (**b**) GB1, (**c**) GB2, and (**d**) GB6 samples.

**Fig. 9:**
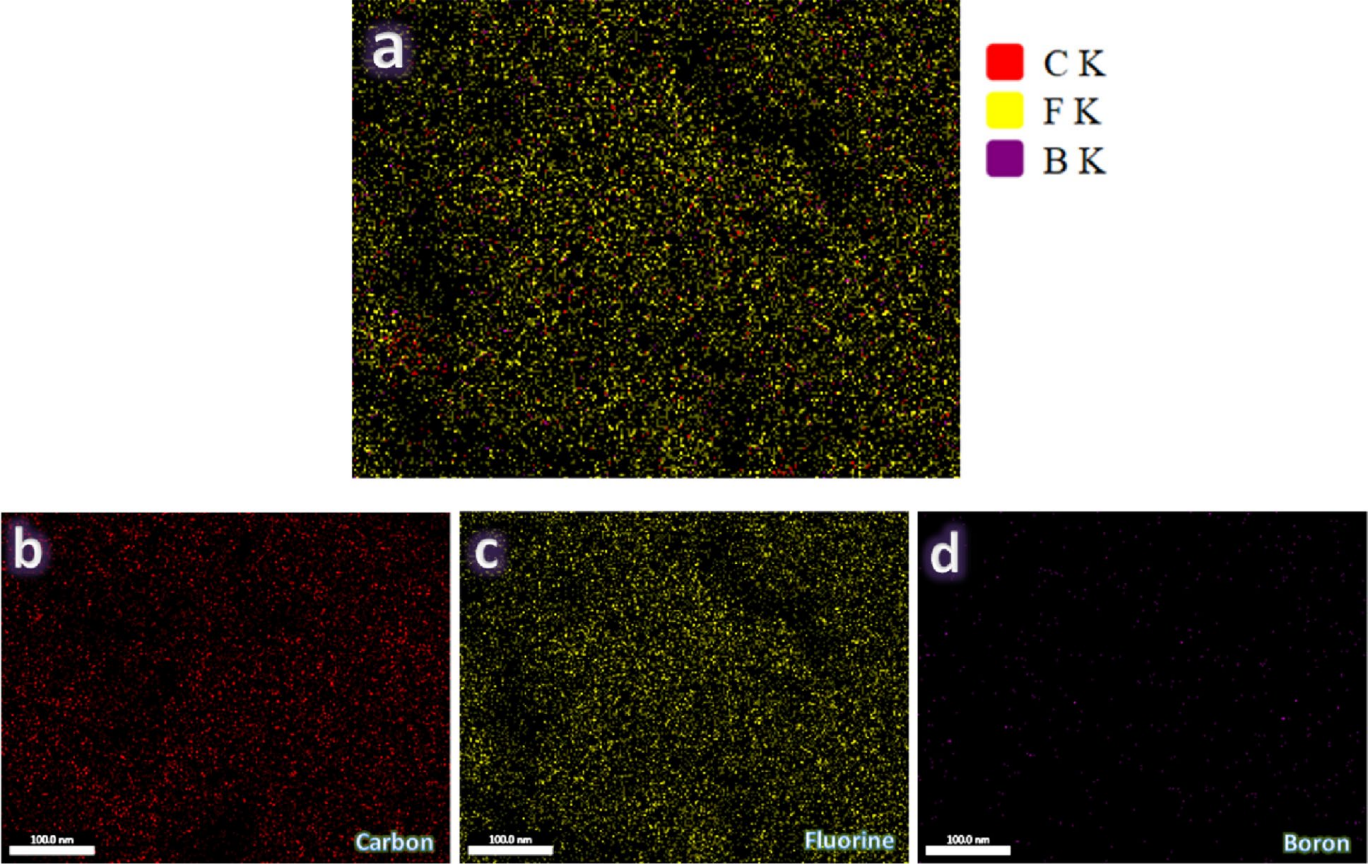
(**a**) EDX mapping of all constituents in the GB6 sample and the EDX mapping of the distribution of each component, i.e., (**b**) carbon, (**c**) fluorine, and (**d**) boron.

Figure 8(a-d) illustrates the microstructure of sintered PTFE and its composites, analyzed by FESEM to assess the influence of graphene and/or B_4_C on the PTFE. Figure 8a depicts microstructure of GB0 sample, characterized by a smooth and uniform surface with few flaws, providing a baseline for comparison. Figure 8b illustrates the microstructure of GB1 sample, revealing little alteration in the PTFE microstructure attributable to the matrix’s modest amount and uniform distribution. Figure 8c, depicting microstructure of GB2 sample, illustrates the uniform distribution of ceramic particles inside the PTFE matrix, characterized by little porosity, robust interfacial bonding, and tiny voids. Figure 8d illustrates the microstuctue of GB6 sample, demonstrating a uniform distribution of both ceramics inside the PTFE matrix, which retains high density and low porosity, signifying enhanced mechanical and wear characteristics. Figure 9(a-d) illustrates an elemental mapping of GB6 sample. As is clear from Fig. 9a, it shows the distribution of all the elements that constitute the sample. Each section of the map illustrates the geographical distribution of specific components, including carbon (Fig. 9b), fluorine (Fig. 9c), and boron (Fig. 9d).

### Thermal properties

**Fig. 10:**
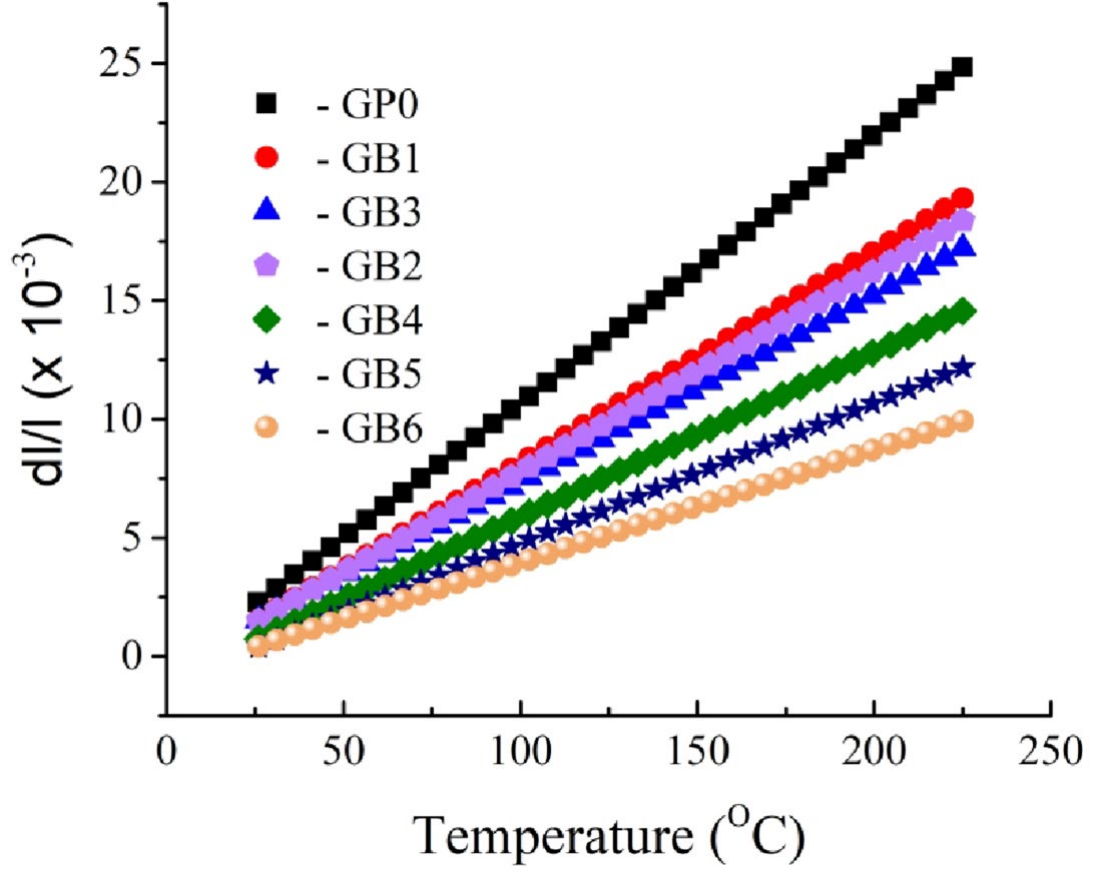
Thermal expansion value of PTFE matrix composites.

**Fig. 11:**
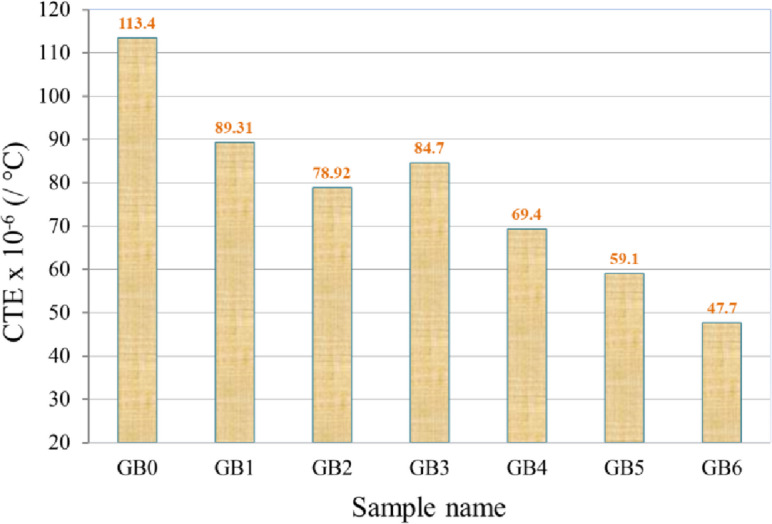
CTE value of PTFE matrix composites.

The relative thermal expansion (dl/l) of samples GB0, GB1, GB2, GB3, GB4, GB5, and GB6 from 25 °C to 225 °C is shown in Fig. 10. The picture illustrates that when the temperature increases, the relative expansion of all samples escalates, albeit it subsequently decreases with the addition of reinforced particles. The CTE for the PTFE matrix and its composites is derived from the preceding figure shown in Fig. 11. The picture illustrates a significant reduction in the CTE value of the PTFE matrix after incorporating graphene and/or B_4_C. The CTE values for the composite samples GB1, GB2, GB3, GB4, GB5, and GB6 are 89.3 × 10⁻⁶, 78.9 × 10⁻⁶, 84.7 × 10⁻⁶, 69.4 × 10⁻⁶, 59.1 × 10⁻⁶, and 47.7 × 10⁻⁶/°C, respectively, reflecting decreases of approximately 21.25, 30.42, 25.31, 38.80, 47.88, and 57.94 compared to the CTE value of the GB0 sample (113.4 × 10⁻⁶/°C). The considerable decrease in the CTE value of the PTFE matrix after incorporating reinforcements can be attributed to the notable disparity between the CTE values of the graphene sheet and B_4_C nanoparticle, approximately 3.2 × 10⁻⁶ and 5.3 × 10⁻⁶/⁰C, respectively, compared to the CTE value of the PTFE matrix, which is less than or equal to 110 × 10⁻⁶/⁰C. Conversely, the reduction in CTE value signifies an enhancement in the interfacial bonding between the PTFE matrix and ceramics; an increase in the movement of molecular chains within the composites becomes restricted due to the presence of reinforcements (steric resistance) and a blockage of molecular chain movement under external heat or force, resulting in stress transfer that diminishes sample expansion and augments mechanical and tribological properties^26,39,40^.

### Mechanical and elastic properties

**Fig. 12:**
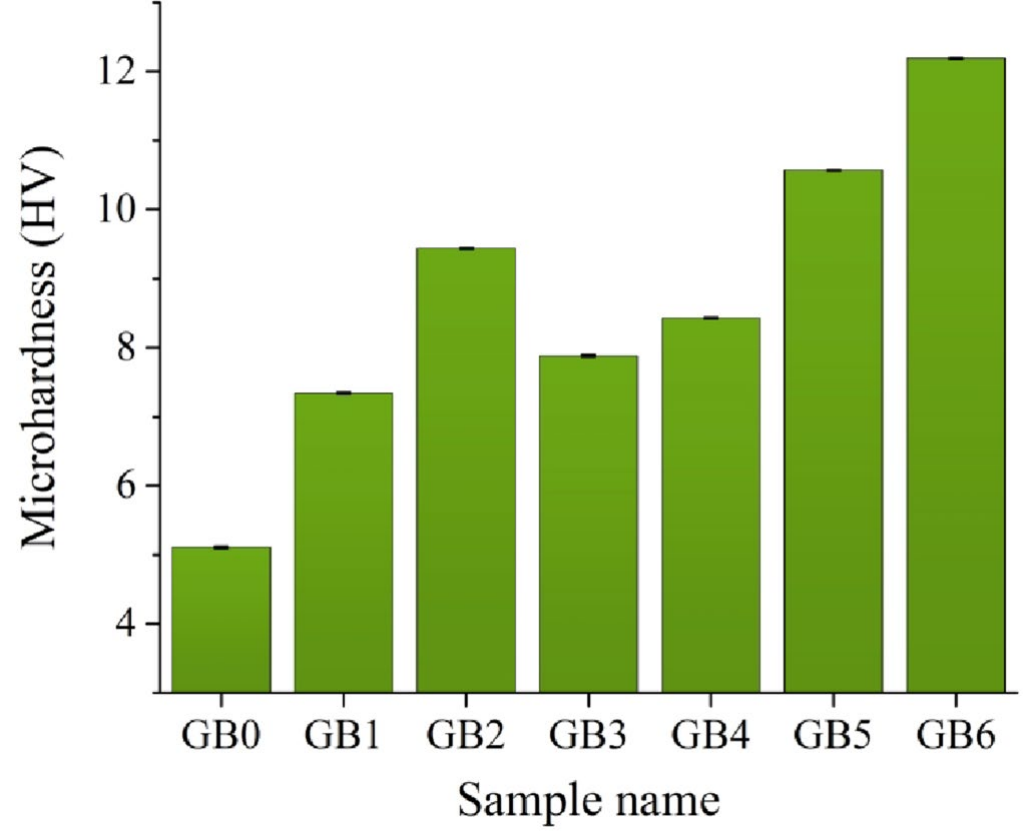
Effect of grapheme or/and B_4_C reinforcement on microhardness of composite samples.

**Fig. 13:**
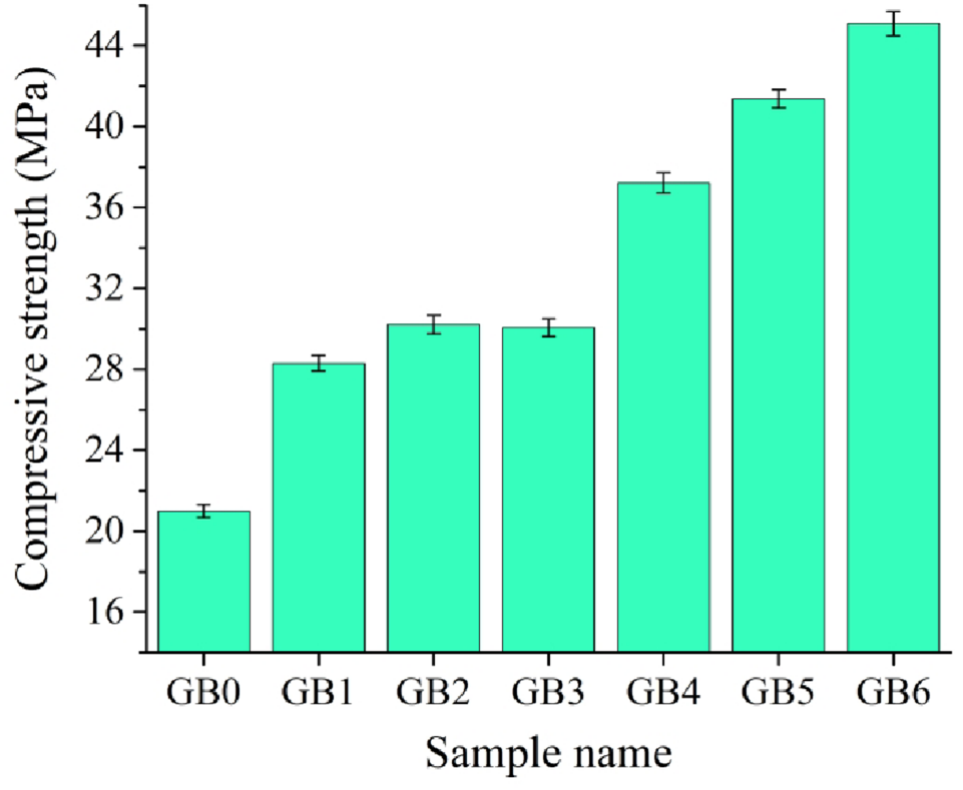
Effect of grapheme or/and B_4_C reinforcement on compressive strength of composite samples.

Figures 12 and 13 illustrate the impact of graphene sheets and/or B_4_C nanoparticles on PTFE’s microhardness and compressive strength after heating to 370 °C for one hour. The microhardness and compressive strength of GB0 sample are 5.11 HV and 20.98 MPa, respectively. After adding mono graphene sheets and B_4_C nanoparticles (GB1 and GB2), the microhardness rises to 7.34 and 9.44 HV, while the compressive strength increases to 28.30 and 30.21 MPa, respectively. The composite samples reinforced with hybrid graphene sheets and B_4_C nanoparticles, GB3, GB4, GB5, and GB6, exhibited improved microhardness values of 7.88, 8.43, 10.57, and 12.19 HV, and compressive strength values of 30.07, 37.22, 41.37, and 45.09 MPa, respectively.

**Fig. 14:**
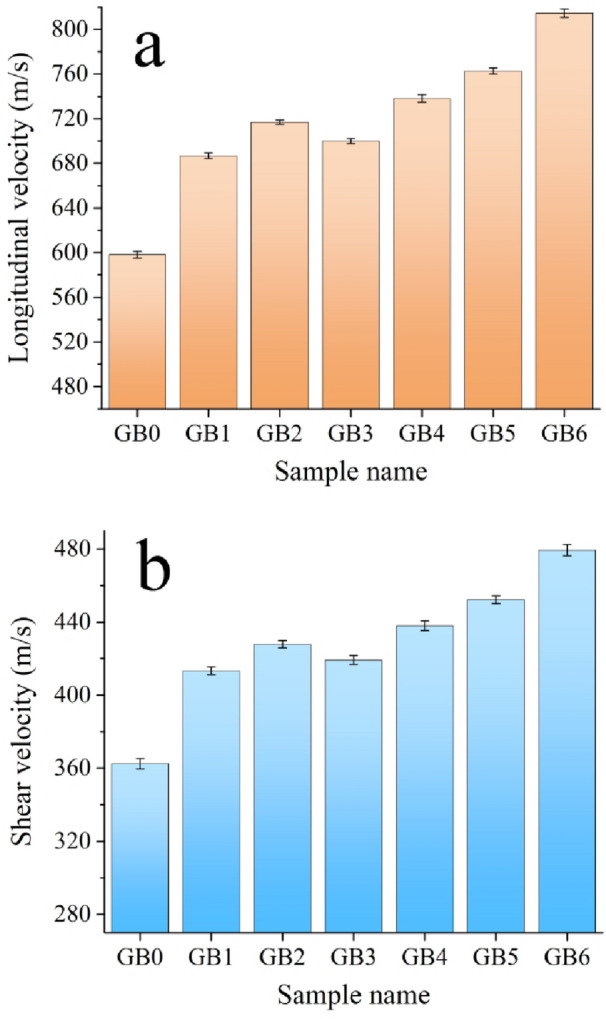
(a) Longatudonal velocity, and (**b**) shear velocity of GB0, GB1, GB2, GB3, GB4, GB5, GB6 samples.

**Fig. 15:**
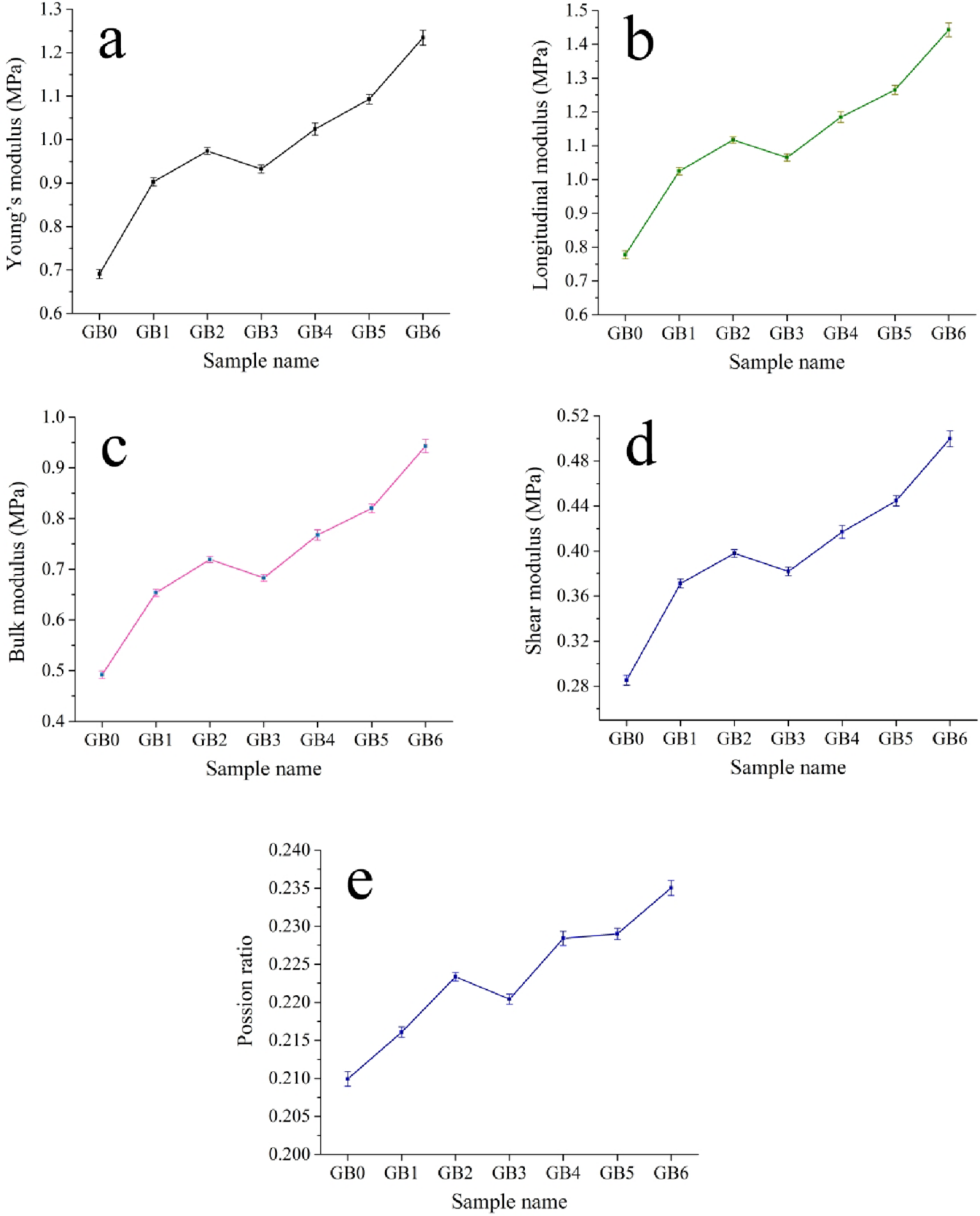
(**a**) Young’s modulus, (**b**) longadudonal modulus, (**c**) bulk modulus, (**d**) shear modulus, and (**e**) possion ratio of GB0, GB1, GB2, GB3, GB4, GB5, GB6 samples.

The group of elastic modulili is a fundamental physical characteristic of engineering materials, signifying the material’s ability to withstand deformation. The longitudinal and shear ultrasonic velocities of PTFE and its composites, obtained using ultrasonic techniques, are shown in Fig. 14, while the elastic moduli are illustrated in Fig. 15. The image demonstrates that longitudinal and shear velocities and elastic moduli exhibit similar behavior to microhardness and strength, substantially increasing their values by incorporating mono and hybrid graphene sheets and B_4_C nanoparticle reinforcements. The longitudinal modulus values for GB0, GB1, GB2, GB3, GB4, GB5, and GB6 are 0.78, 1.01, 1.12, 1.05, 1.75, 1.24, and 1.43 GPa, respectively, whereas the shear modulus values for the same samples are 0.29, 0.37, 0.40, 0.38, 0.41, 0.44, and 0.49 GPa, respectively. The significant enhancement in the mechanical and elastic characteristics of composite samples relative to the PTFE base may be attributed to the reinforcing action of rigid reinforcements and their uniform distribution obstructs dislocation movement, improving Orowan strengthening^41,43,44,44^. Additionally, the reinforcing particles transfer loads efficiently from the PTFE base due to strong interfacial bonding, increasing their resistance to plastic deformation under an external load^45,46^. Moreover, the homogenous distribution of reinforcements leads to a more sophisticated and uniform microstructure, significantly improving the material’s overall performance. This homogeneity inhibits particle aggregation, which may otherwise serve as vulnerabilities within the matrix, resulting in structural discrepancies.Consequently, the material demonstrates more uniform and dependable mechanical properties over its whole volume^32,47,48^. These results are significantly aligned with those recorded in Refs^49,51,51^.

### Tribological properties

**Fig. 16:**
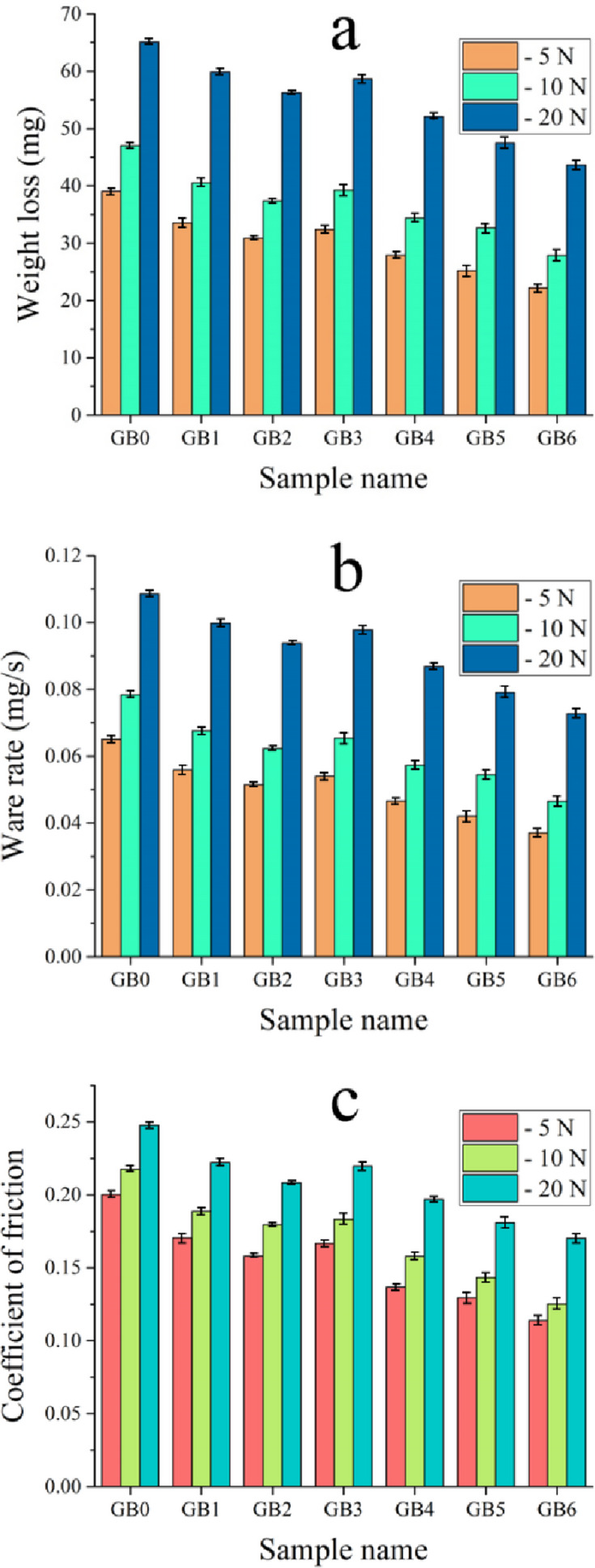
(**a**) Weight loss, (**b**) wear rate, and (**c**) coefficient of friction of all prepared samples.

Figure 16 illustrates the results of the examination into the tribological characteristics of PTFE and its composites under varying loads. The original PTFE (GB0) exhibits low wear resistance due to its inadequate intermolecular cohesion and ability to generate a thin transfer layer on the counterbody^52^. Using mono and hybrid ceramics reduces weight loss, wear rate, and coefficient of friction in all composites. Conversely, the augmentation of the applied load adversely impacts the wear resistance of both PTFE and the composites; as the weight increases, so does the wear rate and coefficient of friction for the samples. The wear rate and coefficient of friction for the GB0 sample under a load of 5 N are 0.0650 mg/s and 0.2001, respectively. Upon raising the load to 10 N, the values escalate to 0.0784 mg/s and 0.2174, rising to 0.01085 mg/s and 0.2474 at a load of 20 N. The ware rates of composite samples (GB1, GB2, GB3, GB4, GB5, GB6) under an applied load of 10 N are 0.0675, 0.0623, 0.0654, 0.0573, 0.0544, and 0.0465 mg/s, respectively. The corresponding friction coefficients for these samples are 0.1887, 0.1795, 0.1832, 0.1579, 0.1432, and 0.1251, respectively. The tribological properties of a material are affected by the phenomena occurring at the surface layers while sliding^53^. Thus, the events happening on the material’s surface during friction substantially impact the coefficient of friction and particular wear rate more than its volumetric properties. The improvement in the wear resistance of PTFE base after added reinforcements is due to a decrease in the propagation of subsurface fractures, a reduction in the size of wear particles, and the formation of transfer films on the surface of the counterbody^54,55^. Furthermore, there is a reduction in the coefficient of friction and an increase in abrasion resistance when reinforced ceramics have a high hardness and thermal stability. The hard reinforcement particles act as load-bearing elements that restrict the plastic deformation of the soft PTFE base and reduce direct contact between the sliding surfaces^56,58,58^. The significant relationship between the increase in the microhardness of the composite based on the PTFE matrix and the decrease in wear rate and coefficient of friction is illustrated by observing the Archard Eq. 16^59^.

16 $$\:\mathrm{W}\mathrm{R}=\mathrm{K}\:\frac{\mathrm{P}}{\mathrm{H}\mathrm{v}}$$

WR is the wear rate, K is the wear coefficient, P is the applied load, and Hv is the microhardness of the sample.

On the other hand, with the increase in load, contact pressure and temperature escalated, resulting in plastic deformation and material loss. This was ascribed to heightened delamination, frictional heat compromising the reinforcement-matrix adhesion, and increasing temperatures softening the matrix. The inclusion of reinforcement particles facilitated the formation of a mechanically mixed layer, hence diminishing wear by decreasing direct interaction with abrasive particles^47,60,62,63,63^. These findings align with several prior studies^45,47–65,65^ examining the impact of ceramics on the wear resistance of PTFE.

### Electrical conductivity

**Fig. 17:**
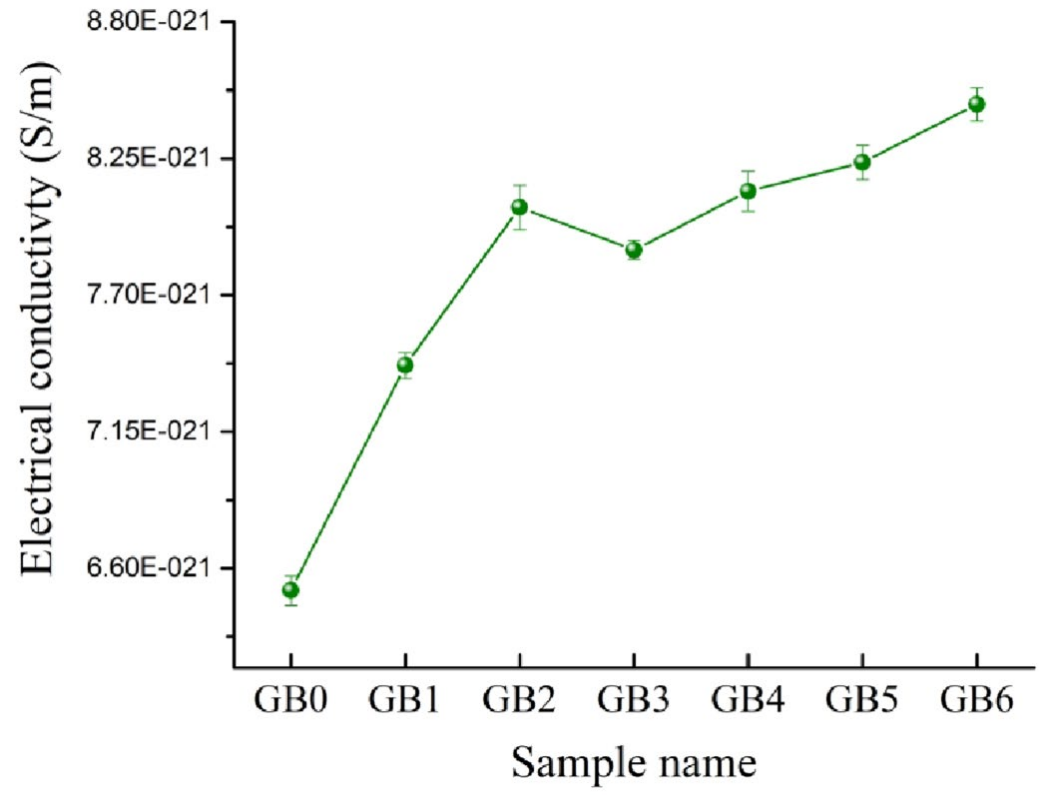
Electrical conductivity of GB0, GB1, GB2, GB3, GB4, GB5, GB6 samples.

Figure 17 illustrates the impact of mono and hybrid B_4_C nanoparticles, as well as graphene nanosheets, on the electrical conductivity of the PTFE matrix. The incorporation of reinforcements results in enhancement of the conductivity values. The electrical conductivity of GB0, GB1, GB2, GB3, GB4, GB5, and GB6 sample is 6.51 × 10^− 21^,7.42 × 10^− 21^, 8.05 × 10^− 21^, 7.88 × 10^− 21^, 8.12 × 10^− 21^, 8.23 × 10^− 21^, and 8.47 × 10^− 21^ S/m, respectively. The reason for the improvement in the electrical conductivity of the PTFE matrix after adding reinforcements is that the electrical conductivity of graphene and B_4_C (~ 10^6^,and 10^5^ S/m, repectivly) is greater than the electrical conductivity of the PTFE matrix (~ 10^− 21^ S/m). These results are consistent with previous articles that the electrical conductivity of PTFE improved after the addition of some ceramics and graphene^66,67^.

## Conclusions

In this research, composites based on PTFE waste are prepared using the Powder metallurgy technique and reinforced with B_4_C nanoparticles and single and hybrid graphene nanosheets. The following findings are the study’s conclusion:

- During the preparation process, the composite material’s content remained unaltered at sintering temperatures of 370 °C and an appropriate pressure of 120 bar, as demonstrated by the XRD technique.
- Adding reinforcements results in a minor alteration in PTFE’s bulk density, total porosity, and relative density. This is because the density of graphene and B_4_C is slightly higher than that of PTFE, which is 2.2 g/cm^3^.
- The CTE of the basic sample is 113.41 × 10⁻⁶/°C, which decreases to 43.50 × 10⁻⁶/°C (about 57.94%) for the GB6 sample, demonstrating that the inclusion of reinforcements considerably influences thermal expansion.
- A notable improvement in the integrated reinforcements and manufactured composites’ mechanical qualities. Compared to the GB0 sample, the GB6 sample had maximum values for microhardness, compressive strength, and Young’s modulus, which were around 1.38, 1.44, and 78.70 times higher, respectively.
- Reinforcements improve the prepared samples’ resistance to wear, although increased applied load negatively impacts wear resistance. In contrast to GB0 with ceramic reinforcements, the wear rate of the GB6 sample decreased by around 43.07%, 40.74%, and 33.01% for applied loads of 5, 10, and 20 N.
- After adding graphene and B_4_C reinforcements, PTFE’s electrical conductivity significantly improved.

## Data Availability

The datasets generated and/or analyzed during the current study are not publicly available because all data are presented in the article and therefore, there is no need to include raw data but they are available from the corresponding author upon reasonable request.
